# Dietary habits and selenium, glutathione peroxidase and total antioxidant status in the serum of patients with relapsing-remitting multiple sclerosis

**DOI:** 10.1186/1475-2891-13-62

**Published:** 2014-06-18

**Authors:** Katarzyna Socha, Jan Kochanowicz, Elżbieta Karpińska, Jolanta Soroczyńska, Marta Jakoniuk, Zenon Mariak, Maria H Borawska

**Affiliations:** 1 Department of Bromatology, Medical University of Bialystok, Mickiewicza 2D St, Bialystok 15-222, Poland; 2 Department of Neurosurgery and Invasive Neurology, Medical University of Bialystok, Bialystok, Poland; 3 NZOZ Kendron, Bialystok, Poland

**Keywords:** Multiple sclerosis, Selenium, Glutathione peroxidase activity, Total antioxidant status, Dietary habits, Smoking cigarettes

## Abstract

**Background:**

Dietary habits and adequate dietary intake of antioxidants in the diet may be one of the most important environmental factors for the prevention of Multiple Sclerosis (MS).

**Objectives:**

The aim of this study was to estimate selenium (Se) concentration, glutathione peroxidase (GSH-Px) activity and total antioxidant status (TAS) in the serum of patients with MS and the influence of dietary habits on the status.

**Methods:**

101 patients with relapsing-remitting MS (aged 18-58 years), as well as control group of 63 healthy people (aged 19-65 years) were studied. Food-frequency questionnaires were implemented to collect the dietary data. Se concentration in the serum samples was determined by atomic absorption spectrometry. GSH-Px activity and TAS in examined serum was measured using the ready-made sets of tests by Randox Laboratories Ltd., UK.

**Results:**

Serum Se concentration and GSH-Px activity in the serum of patients with MS (55.2±16.2 μg/L, 6676.1±2386.4 U/L; respectively) were significantly decreased (p<0.01, p<0.05; respectively) compared with control group (79.2±20.6 μg/L, 8029.9±2650.1 U/L; respectively). A significant correlation (r=0.39, p<0.01) was observed between Se concentration and GSH-Px activity in the serum of examined patients. TAS value in the serum of patients with MS (1.03±0.37 mmol/L) was also significantly lower (p<0.01) than in healthy volunteers (1.48±0.41 mmol/L). Frequent consumption of poultry, bakery products, pulses and fish seemed to increase serum Se concentration in the group of patients; whereas frequent consumption of butter, wholegrain bread, sweet beverages and sugar was found to accompany with lower values of Se in the serum. We have observed significant decrease TAS (p<0.05, p<0.01; respectively) in the serum of smokers and those patients who received immunomodulatory drugs (0.95±0.39 mmol/L, 0.92±0.34 mmol/L; respectively) compared with no-smoking patients and not taking immunomodulators (1.14±0.33 mmol/L, 1.31±0.31 mmol/L; respectively).

**Conclusions:**

Serum Se concentration, GSH-Px activity and TAS value were significantly lower in patients with relapsing-remitting MS compared with healthy volunteers. Dietary habits have a significant influence on Se status. Smoking cigarettes and intake of immunomodulatory drugs therapy have a negative impact on TAS of examined patients.

## Background

Multiple Sclerosis (MS) is an inflammatory demyelinating disease of the central nervous system that may result in a wide range of neurological symptoms and accumulating disability. The exact pathophysiology of MS is not clear but it seems to be autoimmune in nature [1]. Haider et al. have demonstrated an important role of oxidative damage in the pathogenesis of demyelination and neurodegeneration in MS [2]. According to some data also diet can contribute to the development of MS – high-calorie, high-protein, high in fat and sugars can promote MS, whereas low-calorie can reduce the risk [3,4].

Poor dietary selenium (Se) intake and status have been shown to be associated with an elevated risk for various diseases. Key role of Se in human metabolism is attributed to its presence in the glutathione peroxidase (GSH-Px) – an antioxidant enzyme which protects cells against harmful effects of free radicals. Se has an immunostimulant and anti-inflammatory effect [5] and it is highly dependent on dietary sources. The north-eastern region of Poland is an area of low Se concentration in soil, and the population is subjected to a particularly high risk of low Se status [6]. All antioxidants in the body (both enzymatic and non-enzymatic) form a total antioxidant status of an individual. Miller et al. defined total antioxidant status (TAS) as the sum of endogenous and food derived antioxidants of the extracellular fluid of an individual. Cooperation of all different antioxidants provides greater protection against reactive oxygen and nitrogen radicals than any single compound alone [7]. The aim of this study was to evaluate serum Se concentration, GSH-Px activity, and TAS value of patients with MS as well as the influence of dietary habits and smoking cigarettes on the status.

## Methods

One hundred and one patients with relapsing-remitting MS between the ages of 18 – 58 (with an average age of 40.86 ± 10.2 years) who were under the care of the NZOZ Kendron in Bialystok, were enrolled in the study. Patients eligible for the study were met the McDonald criteria for a diagnosis of MS and had a relapsing clinical course [8,9]. According to a 10-point scale EDSS (Extended Disability Status Scale) in examined patients the median was 3,5 (inter-quartile range 2 – 4 points). The average duration of disease was 5,44 ± 5.2 years (range 1 – 23 years). 65% of patients received immunomodulatory drugs (interferon beta, fingolimod, natalizumab, glatiramer acetate). The blood was drawn in remission and as the recent relapse of the disease has passed from least eight months to six years, patients were not treated with corticosteroids. Control blood samples were drawn from 63 healthy volunteers in comparable age (19–65 years, an average age of 41.12 ± 14.1 years) and gender to the study group. The blood from the control group was collected in the same period as the study group (from 2011 until 2013). Patients and control group data are shown in (Table 1).

**Table 1:** Patients and control group characteristic

**Variable**	**Patients with MS (n = 101)**	**Control group (n = 63)**
Gender (M/F)	37/64	20/43
Age (years) – Mean (range)	40.86 ± 10.2 (18-58)	41.12 ± 14.1 (19-65)
Smoking/No-smoking*	45/56	26/37
Immunomodulatory drugs therapy (yes/no)	52/49	0/63

M – male, F – female; * number of cigarettes: 5-20/daily.

Food-frequency questionnaires were implemented to collect the dietary data. Patients with MS were asked to complete the questionnaire of the Committee of Human Nutrition Science, Polish Academy of Sciences, concerning the frequency of food products consumption. The list of food commodities is consist of 36 food items (white bread, wholegrain bread, sweets, cereal products, grain products, pulses, milk, cottage cheese, other sorts of cheese, meat, poultry, offal, sausages, ham, meat products, bacon, tinned meat, tinned fish, fresh fish, eggs, butter, margarine, vegetable oils, potatoes, processed vegetables, fresh vegetables, fruit, sugar added to beverages, marmalade, honey, soft drinks, beer, wine, vodka, coffee, tea). The consumption frequency of different kind of food was estimated according to the following criteria: frequent consumption was defined as an intake of certain food products from twelve to thirty days per month, except fish, that was eaten four to twelve times a month. Food products eaten less frequently were classified into “sporadic consumption” group [10].

Blood samples were drawn from patients and control group were drawn using the vacutainer system test tubes containing clot activator (Becton Dickinson, France). The samples were allowed to clot within 30 minutes then centrifuged within 10 minutes at approximately 1000 × g. Serum was removed and kept frozen at -20°C. The protocol of the study was approved by the Local Ethical Committee (R-I-002/70/2011) and written consent was taken from every participant.

The concentration of Se in the serum was determined by the electrothermal atomic absorption spectrometry method with Zeeman background correction (Z-2000 instrument, Hitachi, Japan). Determine the concentration of Se, TAS and GSH-Px in the tested samples were taken during the month. Every day, certified reference material of human serum (Seronorm Trace Elements, Serum Level 1, 0903106, Sero AS, Norway) was used to test the accuracy of this method. The results of the quality control analyses corresponded with the reference values. The accuracy of the method was 0.28% and the coefficient of variation was 1.6%. The detection limit of the method was 1.49 μg/L. The Department of Bromatology, Medical University of Bialystok participates in a quality control program for trace element analysis supervised by the National Institute of Public Health – National Institute of Hygiene and the Institute of Nuclear Chemistry and Physics. GSH-Px activity and TAS in the serum was measured using the ready-made sets of tests by Randox Laboratories Ltd. (United Kingdom) and UV – VIS spectrophotometer (Cintra 3030, GBC, Australia).

Statistical analyses were performed using Statistica v.10.0 software. Differences between independent groups were tested by the Mann–Whitney U-test. Correlation was calculated by Spearman rank test. To estimate the influence of dietary habits on the Se status in the examined patients, we used a multiple linear regression analysis.

## Results

Se concentration and GSH-Px activity in the serum of patients with MS (55.2 ± 16.2 μg/L, 6676.1 ± 2386.4 U/L; respectively) were significantly lower (p < 0.01, p < 0.05, respectively) than in the control group (79.2 ± 20.6 μg/L, 8029.9 ± 2650.1 U/L; respectively). We observed correlation (r = 0.39, p < 0.01) between Se concentration and GSH-Px activity in the serum. The TAS in the serum of patients with MS (1.03 ± 0.37 mmol/L) was also significantly lower (p < 0.01) than in the healthy volunteers (1.48 ± 0.41 mmol/L) (see Table 2).

**Table 2:** Content of Se, GSH-Px activity and TAS in the serum of patients with multiple sclerosis (MS) and in the control group

	**Selenium (μg/L)**		**GSH – Px (U/L)**		**TAS (mmol/L)**	
	**Control group**	**MS patients**	**Control group**	**MS patients**	**Control group**	**MS patients**
	**mean ± SD**	**mean ± SD**	**mean ± SD**	**mean ± SD**	**mean ± SD**	**mean ± SD**
	**(n)**	**(n)**	**(n)**	**(n)**	**(n)**	**(n)**
All subject	79.2±20.6	55.2^**#**^± 16.2	8029.9± 2650.1	6676.1*± 2386.4	1.48± 0.41	1.03^**#**^± 0.37
	(63)	(101)	(63)	(101)	(63)	(101)
A) Smoking	87.6±24.4	54.9± 16.2	8548.4± 2221.5	6649.6± 2421.0	1.38± 0.31	0.95*^A/B^± 0.39
	(26)	(45)	(26)	(45)	(26)	(45)
B) No-smoking	79.4± 17.52	55.6±13.5	8536.1± 2919.6	6609.2± 1948.4	1.31± 0.41	1.14± 0.33
	(37)	(56)	(37)	(56)	(37)	(56)
C) Taking ID	-	55.4± 17.8		6911.9± 2480.9		0.92^**#C/D**^± 0.34
		(52)		(52)		(52)
D) No-taking ID	-	58.6± 14.8		6684.5± 2433.1		1.31± 0.31
		(49)		(49)		(49)

SD – standard deviation, n – number of subjects, ID - immunomodulatory drugs; p value, p<0,05 - *, p<0,01 - ^**#**^.

Table 3 shows the stepwise multiple linear regression analysis of the influence of frequent consumption of food products on Se concentration in the serum of patients with MS. Frequent consumption of poultry, bakery products, pulses and fish seemed to increase serum Se concentration in the group of patients; whereas frequent consumption of butter, wholegrain bread, sweet beverages and sugar was found to accompany with lower values of Se in the serum. The independent variables included in the model accounted for about 59% of the variance.

**Table 3:** Stepwise multiple linear regression analysis of influence of frequency consumption of food products on content of Se in serum of patients with MS, β coefficients and significance of variables entered in the model

**Independent variables**	**β coefficient (SE)**	**Significance level**	**Model R**^**2**^
Poultry	0.520 (0.127)	0.0004	0.59
Bakery products	0.533 (0.137)	0.0006	
White bread	0.687 (0.189)	0.0012	
Pulses	0.326 (0.115)	0.0087	
Fish	0.352 (0.134)	0.0142	
Butter	-0.652 (0.178)	0.0011	
Wholegrain bread	-0.302 (0.115)	0.0143	
Sweet beverages	-0.262 (0.126)	0.0461	
Sugar	-0.267 (0.134)	0.0573	

SE – standard error.

In the control group frequent consumption of offal, milk, beer, bakery products and fruit seemed to increase Se concentration in the serum; while frequent eating of sausages and vegetable oil was found to accompany with lower values of Se in the serum. The independent variables included in the model accounted for about 67% of the variance (see Table 4).

**Table 4:** Stepwise multiple linear regression analysis of influence of frequency consumption of food products on content of Se in serum of control group, β coefficients and significance of variables entered in the model

**Independent variables**	**β coefficient (SE)**	**Significance level**	**Model R**^**2**^
Offal	0.393 (0.100)	0.0005	0.67
Milk	0.347 (0.101)	0.0017	
Beer	0.250 (0.097)	0.0151	
Bakery products	0.323 (0.132)	0.0203	
Fruit	0.245 (0.115)	0.0419	
Sausages	-0.352 (0.112)	0.0039	
Vegetable oil	-0.396 (0.128)	0.0044	

SE – standard error.

We have observed significantly lower TAS (p < 0.05, p < 0.01; respectively) in the serum of smokers and those patients who received immunomodulatory drugs (0.95 ± 0.39 mmol/L, 0.92 ± 0.34 mmol/L; respectively) compared to no-smoking patients and not taking immunomodulators (1.14 ± 0.33 mmol/L, 1.31 ± 0.31 mmol/L; respectively).

## Discussion

Free radical attack has been linked to numerous pathological conditions such as inflammation and infections in all organs of the body. Reactive oxygen species are generated endogenously during inflammation and lipid peroxidation. Low antioxidant level affects the immune system. Oxidative stress is an important factor involved in the pathogenesis of MS, including speeding up the production of reactive oxygen species [11-13]. Both demyelination and inflammation are linked with the generation of reactive oxygen species. Factor facilitating the activation of oxidative process induced by reactive oxygen species is an increased demand for oxygen by neurons in the central nervous system and decreased concentrations of endogenous antioxidants and other compounds that can inactivate free radicals [12]. Se is an active component of various enzymes involved in redox reactions which protect membranes from oxidative damage. Deficit of selenium is one of the risk factors that may predispose to inflammatory diseases [14]. Dietary Se, mainly through its incorporation into selenoproteins, plays an important role in inflammation and immunity processes so, appropriate concentration of Se is important for immunity but it also helps in regulation of excessive immune responses and chronic inflammation [15].

The reference level of Se in the serum is 70 – 140 μg/L [16] and the reference range of GSH-Px is 4171 – 10881 U/L [17]. We observed decreased Se concentration in the serum of the examined patients with MS, whereas the average level of Se was within the reference range in the control group. Serum concentration of TAS in patients with MS was also below the references ranges for European population (1.30 – 1.77 mmol/L) [17].

So far, the concentration of Se, GSH-Px activity and TAS in the serum of patients with MS have not been estimated in the north-eastern region of Poland. Similar results for the decreased Se concentration in Mongolian patients with MS obtained Komatsu et al. [18]. Also similar to our research, studies of Mai et al. [19] showed lower level of GSH-Px in MS patients than in healthy volunteers. Miller et al. [20] evaluated the TAS in patients with MS living in the central Poland and also found its significant reduction in comparison to the control group.

The content of Se in a diet depends on Se availability in the environment. It has been well documented that Se levels in subjects from different regions of Poland are rather low [21,22]. Our previous investigations showed that a degree ratio of realization of the recommended daily allowance for Se (60 and 70 μg/day for women and men, respectively) was about 50% [23]. A stepwise multiple linear regression analysis showed that dietary habits influence on concentration of Se in the serum both in patients with MS and the control group, but other products have an impact on that status, which may result from differences in the diet of the study groups. An analysis of the frequency distribution of consumption in individual food groups, indicated that diet differs between the patients with MS and the healthy subjects. People with MS more often consumed processed products such as canned meat and canned fish, margarine, sweetened beverages, jam and sugar, while those in the control group frequently consumed products such as fish, fruits, vegetables, legumes. According to the food pyramid, cereals which form the basis were consumed with similar frequency in both groups. Certain cereal products and vegetables are also known to deliver a significant amount of Se to the organism. Among Spanish population levels of Se were positively correlated with the consumption of fruit and vegetables [24]. Among the food stuffs high content of Se is in Brasil nuts, fish, kidney, liver, chicken, eggs and bean [24,25]. Wholegrain cereals, despite the high mineral content, have a lower bioavailability due to a competition between mineral elements in the stage of absorption in the gastrointestinal tract. Additionally, the presence of dietary fiber has a negative impact on bioavailability [26]. In our region, meat products can be a main source of Se in diets. Our earlier study of food products from the Podlasie region showed that meat, especially poultry and offal, are the best source of Se in diets [24,27]. Our earlier research showed that meat products can provide on average about 24% general content of Se in diet [28]. Milk products are rather poor source of Se in our region, but it is considered that Se derived from high protein products has a good bioavailability [29,30]. Our research showed that consumption of sugar and sweet beverages may decrease the serum level of Se. Refined sugar is devoid of minerals, and it is considered that sugar can leach minerals from the body [31]. That explains in a part the results of our work on the influence of dietary habits on serum Se concentration of the patients with MS.

## Conclusions

Serum Se concentration, GSH-Px activity and TAS value were significantly lower in patients with relapsing-remitting MS compared with healthy volunteers. Dietary habits have a significant influence on Se status. Smoking cigarettes and intake of immunomodulatory drugs therapy have a negative impact on TAS of examined patients.

## Competing interests

The authors declare that they have no competing interests.

## Authors’ contributions

KS participated in writing the manuscript, designing the study and took part in the experiments procedures. JK collected the samples and diet questionnaires and took part in clinical consultation. EK estimated samples, performed the statistical analysis and assisted in writing the manuscript. JS estimated samples. MJ and ZM participated in the design of the study and took part in clinical consultation. MHB participated in study coordination and in manuscript preparation. All authors read and approved the final manuscript.

## Authors’ information

Elżbieta Karpińska who “Studies, research, commercialization – a support programme of UMB doctoral students” Sub-measure 8.2.1 Human Capital Operational Programme, co-financed from the European Union under the European Social Fund.
